# Impact of *c-MYC* expression on proliferation, differentiation, and risk of neoplastic transformation of human mesenchymal stromal cells

**DOI:** 10.1186/s13287-019-1187-z

**Published:** 2019-03-05

**Authors:** Svitlana Melnik, Nadine Werth, Stephane Boeuf, Eva-Maria Hahn, Tobias Gotterbarm, Martina Anton, Wiltrud Richter

**Affiliations:** 1 Research Center for Experimental Orthopaedics, Center for Orthopaedics, Trauma Surgery and Paraplegiology, Heidelberg University Hospital, Schlierbacher Landstrasse 200a, 69118 Heidelberg, Germany; 2 Department of Orthopedics, Kepler University Hospital, Linz, Austria; 3 Institutes of Molecular Immunology and Experimental Oncology, Klinikum rechts der Isar, Technical University Munich, Munich, Germany

**Keywords:** MYC, Mesenchymal stromal cells (MSC), Osteogenesis, Adipogenesis, Chondrogenesis, Tumorigenesis, P14ARF, P16INK4A, CDKN2A

## Abstract

**Background:**

Mesenchymal stromal cells isolated from bone marrow (MSC) represent an attractive source of adult stem cells for regenerative medicine. However, thorough research is required into their clinical application safety issues concerning a risk of potential neoplastic degeneration in a process of MSC propagation in cell culture for therapeutic applications. Expansion protocols could preselect MSC with elevated levels of growth-promoting transcription factors with oncogenic potential, such as c-MYC. We addressed the question whether *c-MYC* expression affects the growth and differentiation potential of human MSC upon extensive passaging in cell culture and assessed a risk of tumorigenic transformation caused by MSC overexpressing c-MYC in vivo.

**Methods:**

MSC were subjected to retroviral transduction to induce expression of *c-MYC*, or *GFP*, as a control. Cells were expanded, and effects of *c-MYC* overexpression on osteogenesis, adipogenesis, and chondrogenesis were monitored. Ectopic bone formation properties were tested in SCID mice. A potential risk of tumorigenesis imposed by MSC with *c-MYC* overexpression was evaluated.

**Results:**

C-MYC levels accumulated during ex vivo passaging, and overexpression enabled the transformed MSC to significantly overgrow competing control cells in culture. C-MYC-MSC acquired enhanced biological functions of c-MYC: its increased DNA-binding activity, elevated expression of the c-MYC-binding partner MAX, and induction of antagonists *P19ARF/P16INK4A*. Overexpression of *c-MYC* stimulated MSC proliferation and reduced osteogenic, adipogenic, and chondrogenic differentiation. Surprisingly, *c-MYC* overexpression also caused an increased *COL10A1/COL2A1* expression ratio upon chondrogenesis, suggesting a role in hypertrophic degeneration. However, the in vivo ectopic bone formation ability of *c-MYC*-transduced MSC remained comparable to control GFP-MSC. There was no indication of tumor growth in any tissue after transplantation of c-MYC-MSC in mice.

**Conclusions:**

*C-MYC* expression promoted high proliferation rates of MSC, attenuated but not abrogated their differentiation capacity, and did not immediately lead to tumor formation in the tested in vivo mouse model. However, upregulation of MYC antagonists *P19ARF/P16INK4A* promoting apoptosis and senescence, as well as an observed shift towards a hypertrophic collagen phenotype and cartilage degeneration, point to lack of safety for clinical application of MSC that were manipulated to overexpress *c-MYC* for their better expansion.

**Electronic supplementary material:**

The online version of this article (10.1186/s13287-019-1187-z) contains supplementary material, which is available to authorized users.

## Background

While embryonic stem (ES) cells represent pluripotent cells with a capacity to form any cell type and tissue in human body during embryonic development, adult stem cells serve for cell replenishment in case of injury or loss due to limited lifespan of terminally differentiated cells. An important class of adult stem cells are mesenchymal stromal cells (MSC) that have been found in bone marrow and many other postnatal tissues and shown to develop into the cells of various mesenchymal tissues such as fat, cartilage, or bone [1, 2]. MSC from bone marrow and adipose tissue are an attractive cell source for regenerative medicine due to their high proliferation capacity [3], multi-lineage differentiation potential [4], and the trophic support of surrounding tissues through secretion of bioactive factors [5]. Therefore, MSC are not only applied for treatment of degenerative musculoskeletal diseases [6, 7], but also for other stem cell-based therapies, such as steroid-refractory graft-versus-host disease (GvHD) [8], vascular diseases [9], neurological disorders [10], and others [11].

However, only limited numbers of bone marrow-derived MSC, which also decline with age of a donor [12], could be obtained from a patient by painful and invasive isolation techniques. Thus, for successful outcome that requires large cell quantities for many clinical applications, MSC need to be expanded in a long-term cell culture. This, however, raises concerns about clinical safety of MSC that have undergone ex vivo expansion. Although their ability to evade immune surveillance [13] and reported immunosuppressive properties [14] make MSC an ideal tool for clinical applications [15], long culture expansion could impact these characteristics [16].

The major concern for the therapeutic application of MSC is the risk of possible tumorigenic transformation. It has been demonstrated that, upon prolonged ex vivo expansion, MSC may undergo cellular senescence [3, 17]. Even though no apparent accumulation of chromosomal aberrations or genetic mutations have been reported for MSC that undergone senescence due to prolonged ex vivo passaging [3, 18], epigenetic changes associated with senescence, differentiation and alteration of immune response of these cells have been described [19, 20]. The question whether MSC could undergo malignant transformation themselves or induce tumor growth remains controversial, as there have been reports ruling out a possibility of malignant transformation of MSC [21–25], as well as studies demonstrating that these concerns cannot be dismissed [26]. In respect of tumor pathogenesis, MSC could either promote and assist cancer cell growth [27] or undergo neoplastic transformation themselves [28–30]. In October 2011, the Cell Products Working Party (CPWP) arranged a meeting between the experts working in the MSC research field to discuss contradictory finding on this subject and define certain standards in MSC cultivation and quality tests before their clinical application. It has been concluded that more clinical observations supported by animal studies would be necessary to address the existing concerns about MSC tumorigenicity in clinical applications [31].

Exposure to the growth factors present in culture media for ex vivo expansion could lead to activation of oncogenic transcription factors triggering neoplastic transformation of the MSC. Available protocols designed to achieve high proliferation rates may condition for selection of those MSC that overexpress the growth-promoting factors. It has been shown that serum growth factors used in common ex vivo expansion protocols can increase *c-MYC* expression levels and consequently stimulate higher cell growth rates [32]. Growth factors, such as bFGF (basic fibroblast growth factor) [33], PDGF (platelet-derived growth factor) [34], and various BMPs (bone morphogenetic proteins) [35], have been demonstrated to induce *MYC* expression. Additionally, in case of murine bone marrow mesenchymal stem cells (BMSC), their ex vivo expansion resulted in higher expression of *c-MYC* in comparison to the initial cell population [36]. Furthermore, bone marrow MSC-conditioned medium has been demonstrated to promote cancer development via upregulation of *c-MYC* [37]. Therefore, *c-MYC* expression provides and supports high proliferation rates of MSC which are necessary for their expansion for many therapeutics applications.

However, *c-MYC* plays not only an important role in cell proliferation, but also is involved in other multiple functions, such as cell differentiation, apoptosis, cell cycle progression, and cellular transformation leading to tumor pathogenesis. The *MYC* (MYC Proto-Oncogene, BHLH Transcription Factor, other names are *C-MYC* or *V-MYC*) family of proto-oncogenes consists of *c-MYC* that is found to be amplified in many types of cancer, and other paralogs expressed in specialized cases, such as *MYCN* (this gene amplification has been detected only in neuroblastoma [38]), and *MYCL* (has been found in lung carcinoma [39]). All MYC proteins are transcription factors with basic helix loop helix motifs that are required for heterodimerization with MAX (MYC-associated protein X). The MYC/MAX heterodimer binds to E-box DNA recognition elements in the promotor region of target genes causing activation of transcription. In this complex, MAX protein determines E-box specificity, and MYC works as an activator. MAX can additionally form heterodimers with the related proteins of the MAD/MNF family, which in turn antagonize the activating effect of MYC/MAX on the same targets. In many cases, the antagonism between MYC and MAD in vivo can be related to a switch of cells from proliferation (MYC/MAX activation) to differentiation (MAD/MAX repression) [40]. Thus, MAD proteins play an important role in antagonizing MYC function, which could also be relevant in MSC.

Another antagonist of MYC is the tumor suppressor P19ARF that can block activating functions of MYC by direct binding, without affecting its expression [41]. *P19ARF* and *P16INK4A* tumor suppressor genes are both products of a common gene *CDKN2A* (cyclin-dependent kinase inhibitor 2A). They are mediators of cellular senescence and apoptosis and have been shown to antagonize aberrant growth signaling caused by gain-of-function of MYC and RAS proteins [42], in particular, to protect cells from neoplastic transformation. Also, in human MSC, *P16INK4A* expression has been shown to correlate with replicative senescence [43]. Thus, the correlations between MYC and P19ARF/P16INK4A could be a key switching point in the transition from stem cell function to senescence and concomitant loss of stem cell properties of MSC.

Deregulated expression of *c-MYC* has been implicated in progression of many types of cancer. The involvement of MYC for the emergence of carcinogenesis is well documented [44–47], as well as its crucial role in the regulation of pluripotency and self-renewal capacity of murine stem cells: ES, neural (NSC) and hematopoietic (HSC), by using various transgenic mouse models [48]. It is important to mention that *MYC* overexpression itself, without other mutations, is not sufficient for tumorigenic transformation. For example, it has been demonstrated that only 1 in 10 of transgenic mice that have *c-Myc* gain-of-function could develop a tumor, with an average latency period of 200 days. However, when overexpression of *c-Myc* in this animal model was combined with gain-of-function of another oncogene, *Her2* (other name Erbb2: erb-b2 receptor tyrosine kinase 2), tumor penetrance reached 100% [49]. Similar effects have been demonstrated for the cases when *c-MYC* overexpression was combined with a loss or silencing of tumor suppressor genes, e.g., *CDKN2A* locus [50], or with RB (Retinoblastoma) protein inactivation [51], when these led to transformation of MSC and acquisition of a malignant osteosarcoma phenotype.

In contrary to murine stem cells, there are limited data on a role of *MYC* in adult human stem cell populations, such as NSC, HSC, and MSC. This question has been investigated in more details only for human epidermal stem cells [52]. Surprisingly, in these cells, *MYC* rather stimulated cell differentiation than their proliferation. This implies that effects caused by *MYC* expression could be context-dependent and more complex than a simple on/off mechanism. For example, the observed triggering of epidermal stem cell differentiation by *MYC* could be interpreted as a cell safety mechanism against the oncogenic potential of high *MYC* levels [53].

There are only very few reports about a possible correlation between the c-MYC protein levels and the growth potential of human MSC [54, 55]. Namely, it has been shown that in human adipose tissue-derived stem cells, *c-MYC* expression could be enhanced upon ex vivo expansion, and this correlated with increased proliferation rates of adipose tissue mesenchymal stromal cells (ASC) [32]. There are no other direct data on a possible impact of high *MYC* expression on growth, differentiation, and tumorigenic potential of human bone marrow-derived MSC. It yet remains to be assessed how MSC ex vivo expansion would affect their stem cell-like properties and whether these cells would impose a safety risk due to increased tumorigenic potential that might hinder their therapeutic application.

The aim of this study was to provide data on a role of *c-MYC* expression during extensive ex vivo expansion of bone marrow-derived MSC and examine whether *c-MYC* overexpression impacts proliferation and differentiation capacities of these cells. We also assayed how elevated expression of *c-M*Y*C* in MSC would affect their bone tissue formation ability in vivo and whether it might enforce a potential risk of tumor formation in recipients, to address the concerns about consequent implications for therapeutic compatibility of these cells.

## Methods

### Isolation and expansion of MSC

The study with application human donor samples was approved by the local ethics committee (Medical Faculty of the University of Heidelberg), and an informed consent was obtained from all the patients participating in the study, according to the 1964 Declaration of Helsinki, updated in 2000. Only cells from HIV-, HBV-, and HCV-negative donors were used. Human ASC were obtained from liposuction aspirates of a human donor, and isolated, as described before [56]. Articular chondrocytes were isolated from articular cartilage resected from tibia plateaus of a patient undergoing total knee replacement, as described [57]. Bone marrow MSC were isolated from fresh bone marrow aspirates of human donors (*n* = 20) that had undergone a total hip replacement procedure. MSC population was isolated, as described before [58]. In brief, cells were fractionated by Ficoll-Paque™ density gradient centrifugation. The mononuclear cell fraction was seeded at a density of 1.25 × 10^5^cells/ cm^2^ in 0.1% gelatin-coated flasks and maintained at 37 °C in humidified atmosphere with 6% CO_2_. Next day, cells were washed with phosphate-buffered saline (PBS), to remove non-adherent cells, and expanded in ES medium composed of DMEM with high glucose, 12.5% FCS, 2 mM l-glutamine, 50 μM β-mercaptoethanol, 1% non-essential amino acids (all from Gibco, Invitrogen, Karlsruhe, Germany), 100 units/ml penicillin, 100 μg/ml streptomycin, and 4 ng/ml basic fibroblast growth factor (bFGF) (Active Bioscience, Hamburg, Germany). When indicated, bFGF was omitted from ES media for the duration of MSC expansion. For osteogenic or adipogenic differentiation, expanded MSC were harvested at passages 4, 6, 8, and 10 with trypsin/ethylenediaminetetraacetic acid (EDTA). Cells were seeded at densities 3.5 × 10^5^ cells per well into a 24-well cell culture plate with corresponding differentiation media.

### Osteogenic differentiation of MSC

After expansion, MSC were subjected to osteogenic-induction medium consisting of high-glucose DMEM, 10% FCS (Biochrom, Berlin, Germany), 0.1 μm dexamethasone, 0.17 mM ascorbic acid 2-phosphate, 10 mM β-glycerophosphate (all from Sigma, Deisenhofen, Germany), 100 units/ml penicillin, and 100 μg/ml streptomycin. Cells were cultured for 3 weeks with medium changes twice per week. To monitor osteogenic differentiation, cells were stained with 0.5% Alizarin Red S (Chroma, Münster, Germany), to detect calcium deposition. Next, they were treated with 10% cetylpyridiniumchloride-solution (Sigma, Deisenhofen, Germany) to extract calcium-bound dye, and calcium content was measured using spectrophotometry at 570 nm. The values were normalized to the total protein content in cell lysates using Bradford Reagent (Sigma, Deisenhofen, Germany).

### Adipogenic differentiation of BMSC

After expansion, MSC were subjected to adipogenic induction medium consisting of DMEM high glucose, 10% FCS, 1 μm dexamethasone, 0.2 mM indomethacine, 0.5 mM isobutylmethylexanthine (all Sigma, Deisenhofen, Germany), 0.01 mg/ml insulin (Sanofi-Aventis, Frankfurt, Germany), 100 units/ml penicillin, and 100 μg/ml streptomycin. Cells were cultured for 3 weeks with medium changed twice per week. To monitor adipogenesis, cells were fixed with 4% paraformaldehyde and stained with 0.3% Oil Red O solution (Chroma, Münster, Germany). Dye was re-extracted from vacuoles by 60% isopropanol and quantified by measuring its optical density at 490 nm.

### Chondrogenic differentiation of MSC

For chondrogenic differentiation, MSC were harvested after passage 4, and 5 × 10^5^ of cells were collected in 1.5-ml Eppendorf tubes by centrifugation (600*g*, 10 min), and subjected to high-density 3D culture in chondrogenic induction medium containing high-glucose DMEM supplemented with 0.1 μM dexamethasone, 0.17 mM ascorbic acid 2-phosphate, 5 μg/ml transferrin, 5 ng/ml selenous acid, 1 mM sodium pyruvate, 0.35 mM proline, 1.25 mg/ml BSA, 100 units/ml penicillin, 100 μg/ml streptomycin, 5 μg/ml insulin (Sanofi-Aventis, Frankfurt, Germany), and 10 ng/ml TGF-β1 (Peprotech, Hamburg, Germany). Pellets were cultured up to 6 weeks, with medium changed three times per week. To monitor chondrogenic differentiation, proteoglycan deposition was measured. For this, pellets were fixed with 4% paraformaldehyde, embedded in paraffin, and 5-μm sections were cut and stained with Safranin O solution (Safranin T Fluka Nr. 84,120, Fluka, Monte Carlo) and Fast Green (Chroma 1A 304, Chroma, Münster, Germany).

### Quantification of proteoglycan content (DMMB assay)

Proteoglycan content in cartilaginous tissue was measured by DMMB (dimethyl-methylene Blue) assay. For this, pellets (*n* = 2 per donor) were harvested at day 42 of the chondrogenic induction, washed with PBS, and digested overnight in 1 ml of lysis buffer containing 50 mM Tris, pH 8.0, and 1 mM CaCl_2_ with 500 μg/ml Proteinase K (Roche, Mannheim, Germany) at 60 °C. Thirty microliters of digested pellets were mixed with 200 μl of DMMB solution (38 μM dimethyl-methylene blue, Sigma, 40 mM glycine, 40 mM NaCl), and proteoglycan content was measured by spectrophotometry at 540 nm, and quantified using a standard curve built using chondroitin sulphate as a standard. The values were normalized to DNA amount in lysed cells measured with Quanti-iT PicoGreen dsDNA kit (Invitrogen, Eugene, USA). For this, 20 μl of the digested pellet sample were mixed with 80 μl TE buffer (200 mM Tris HCl, 20 mM EDTA) and PicoGreen solution, and fluorescence in samples was measured at 485/535 nm.

### Retroviral vector cloning and retrovirus production

For retroviral expression of *MYC*, a 1320 bp fragment encoding human v-myc myelocytomatosis viral oncogene homolog (*MYC*) was sub-cloned from pMXs-hc-*MYC* plasmid (a gift from Shinya Yamanaka, Addgene plasmid # 17220 [59]) into the NcoI site of the retroviral vector pBullet [60], after blunt-ending reaction with Klenow enzyme. The similar approach was taken for cloning of a control construct expressing *eGFP* (enhanced green fluorescent protein). The resulting vectors were designated as pBullet-*cMYC* and pBullet-*eGFP*. For retrovirus production, HEK293T cells were transfected with retroviral vectors (either with pBullet-*cMYC* or pBullet-*eGFP*, together with helper plasmids pHit60 and pHCMV-G, as described before [61]) using calcium phosphate transfection system (Life Technologies, Groningen, The Netherlands), according to the manufacturer’s protocol. In brief, 2 × 10^6^ cells were seeded on 10-cm dishes and transfected the next day using 10 μg of pBullet-*cMYC* or pBullet-*eGFP*. After 18 h, culture medium was discarded and replaced with 5 ml of fresh supplement. The next day, medium containing retrovirus was harvested, filtered through a 0.45 μm filter, and used for retroviral transduction. Retroviruses used for the subsequent transduction experiments were estimated to be at MOI (multiplicity of infection) = 5.

### Retroviral transduction of MSC

After isolation, MSC were passaged 1 day before retrovirus transduction experiment. For this, 450 μl of virus supernatant, 550 μl ES medium, and 8 μg/ml polybrene were mixed and added to culture medium of MSC. Two hours later, additional 4 ml of ES medium were added, and media was replaced 48 h later. Cells were expanded for up to 15 passages at a seeding density of 4000 cells/cm^2^. For differentiation experiments, MSC were transferred to corresponding differentiation media at passage 4, 6, 8, or 10. At every passage, cell numbers and culture time were noted in order to record a cumulative expansion time cell numbers.

### RNA extraction and quantitative mRNA expression analysis (qRT-PCR)

Total RNA was isolated from pellets using a standard guanidiniumthiocyanate/phenol extraction protocol (peqGOLD TriFastTM; Peqlab, Erlangen, Germany). Polyadenylated mRNA was isolated using oligo d(T)-coupled magnetic beads (Dynabeads, Dynal, Invitrogen GmbH, Karlsruhe, Germany) according to the manufacturer’s instruction. Twenty nanograms of mRNA was used for the first strand cDNA synthesis with reverse transcriptase (Omniscript®, Qiagen, Hilden, Germany) and oligo-d(T) primers. Quantitative reversed transcriptase PCR (qRT-PCR) was performed using SYBR green I mix (Thermo Scientific, Rockford, USA) and gene-specific primers (Additional file 1: Table S1) with StratageneMx3000P (Agilent Technologies, Böblingen, Germany). mRNA expression was calculated using 2^−ΔΔCT^ method [62], with β-actin (*ACTB*) used as a reference gene.

### Western blot (WB) analysis

Cells from pellets were lysed in lysis buffer containing 50 mM Tris 7.4, 150 mM NaCl, 1% Triton X-100 for 5 min on ice. Lysates were cleared by centrifugation and proteins were resolved by SDS-PAGE, blotted onto a nitrocellulose membrane, and analyzed by Western blotting (WB). The following antibodies were used: c-MYC (clone 9E10, SC-40, Santa Cruz) and β-actin (AC-15, GeneTex, USA) was used as reference protein.

### MYC DNA-binding activity ELISA assay

DNA-binding activity of c-MYC was measured with TransAM c-Myc Transcription Factor ELISA Assay Kit (Active Motif, Inc., Carlsbad, USA), according to the manufacturer’s instructions. In brief, 2.5 μg of nuclear extracts isolated from cell pellets with Nuclear Extract Kit (Active Motif, Inc., Carlsbad, USA) were incubated with a synthetic oligonucleotide containing the c-MYC consensus sequence. After incubation, DNA-bound c-MYC was detected with c-MYC antibody and HRP-conjugated secondary antibody by a colorimetric assay, and quantified using a standard curve. Nuclear extract from Jurkat cells, as well as positive and negative controls for binding with c-MYC (oligonucleotides containing either mutated or wild-type c-MYC-binding sequence, respectively, in competition with the tested samples) were used as assay controls (data not shown).

### Animal experiments

Animal care and all animal experiments were performed according to the national guidelines, approved by the responsible national authority, the local Governmental Committee for Animal Experimentation (Regierungspräsidium Karlsruhe, Germany), and carried out accordingly.

### Preparation of β-TCP constructs

Beta-tricalcium phosphate hydrate (β-TCP) constructs [63] were prepared as described before [56]. In brief, β-TCP grains (10 mg, particle size 0.25–1 μm, Sigma-Aldrich Chemie GmbH, Steinheim, Germany) were autoclaved in 30 μl phosphate-buffered saline (PBS), to preserve hydrophilic properties. After complete removal of PBS, 1 × 10^6^ MSC collected after passage 2 were resuspended in 10 μl of fibrinogen (Tisseel, Baxter, Unterschleissheim, Germany) diluted 1:15 in PBS and added to the β-TCP, together with a short (1.5–2 cm) surgical thread inserted in a tube, then incubated for 10 min. A cell-free β-TCP/fibrinogen and biomaterial-free cells/fibrinogen suspensions were prepared as controls. Next, 10 μl of 1:50 diluted thrombin (Tisseel) were added, to allow polymerization into a β-TCP construct at room temperature for 5 min. After preparation, β-TCP constructs were soaked in 1 ml of PBS dislodged from a tube and implanted into host animals.

### In vivo ectopic bone formation

For ectopic bone formation experiments, female SCID mice, *n* = 8 (CB17/Icr-Prkdcscid/IcrIcoCrl, Charles River, Sulzfeld, Germany) aged at 10–12 weeks were used as hosts. Immediately after preparation, β-TCP constructs were implanted surgically into skin pockets (5 mm × 5 mm) of host animals under general anesthesia (120 mg/kg ketamine (Ketavet®, 100 mg/ml, Pfizer) and 0.5 mg/kg medetomidine hydrochloride (Domitor®, 1 mg/ml, Pfizer)). Up to four constructs per animal, comprising a combination of different β-TCP constructs (*c-MYC*-transduced, *GFP*-transduced, or control MCS constructs, along with control cell-free, and biomaterial-free constructs), were implanted in anterior or posterior parts at a dorsal side of a mouse. After the transplantation, animals were sacrificed at day 42 via CO_2_; the implants were excised and subjected to histological analyses and in situ hybridization assay.

### Histology

Histological evaluation of expanded MSC after differentiation and explanted β-TCP constructs was performed, as described [56]. In brief, β-TCP constructs or MSC pellets were fixed in 4% paraformaldehyde for 24 h. β-TCP constructs were additionally decalcified in ethylenediaminetetraacetic acid (EDTA) for 4–5 days. Next, specimens were dehydrated using a graded alcohol series, embedded in paraffin, sectioned into 5-μm slices, and either stained with hematoxylin-eosin (HE), according to a standard protocol, or subjected to in situ hybridization assay. For histomorphometrical analysis, to quantify a total area of bone tissue, 48–54 sections were analyzed for each treatment group using ImageJ software.

### In situ hybridization assay

To discriminate cells of human or murine origin, in situ hybridization was performed, as described before [64]. In brief, after fixation and cutting, thin sections were subjected to hybridization with DIG-labeled probes. To distinguish between human or mouse cells, probes specific to human genomic *ALU* sequences or to murine genome repetitive elements *Sine/B1* and *Sine/B2* were used, respectively. After hybridization, signals were detected using anti‐DIG alkaline phosphatase‐conjugated Fab fragments (Roche, Germany) and NBT/BCIP (Roche, Germany) as substrate.

### Statistical analysis

Data are presented as mean values ± standard deviation. Statistical analysis was performed using SPSS software (SPSS Inc., Chicago, IL, USA), with application of Mann-Whitney *U* test. For comparison of growth curves, ANOVA test with Bonferroni correction was applied. *P* values < 0.05 were referred to as being significant.

## Results

### Human MSC displayed c-MYC protein accumulation that was increased upon ex vivo passaging

**Fig. 1:**
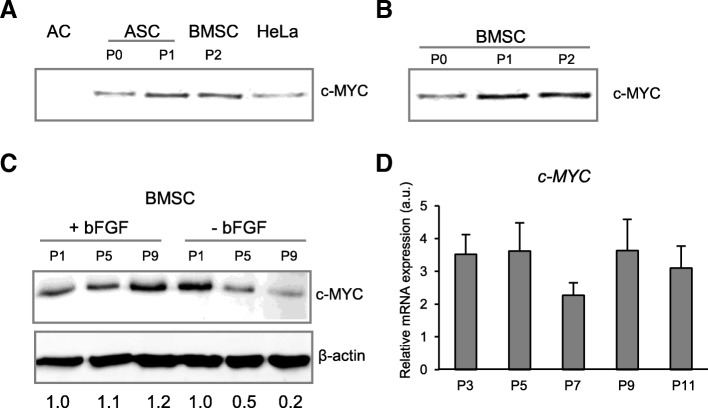
c-MYC protein accumulation that was increased upon ex vivo passaging. **a**, **b** Western blot analysis of c-MYC protein abundancies in different human mesenchymal cell types. Cellular extracts from 25,000 cells for every sample were analyzed by Western blot, with equal volume loading. **a** c-MYC was assayed in freshly isolated articular chondrocytes (AC), MSC from adipose tissue (ASC), bone marrow MSC (BMSC), and Hela cells served as a positive control for c-MYC protein accumulation. **b** Bone marrow-derived MSC isolated at passages 0 (P0), 1 (P1), and 2 (P2). **c**, **d** c-MYC protein accumulation and mRNA expression were monitored by Western blot (**C**) and qRT-PCR (*n* = 3) (**d**) in BMSC at indicated passages (P1, P3, P5, P7, P9, P11), in media with or without basic fibroblast growth factor (bFGF), as indicated (**c**); numbers below WB in C indicate semi-quantitative evaluation of c-MYC protein abundancies normalized to β-actin, as a fold change to a corresponding P1

Due to lack of data available on *c-MYC* expression in human MSC, we first examined the c-MYC protein levels in different types of human mesenchymal cells: articular chondrocytes (AC), adipose tissue-derived MSC (ASC), and bone marrow MSC (BMSC), in comparison to human cervical cancer cell line HeLa serving here as a positive control (Fig. 1a). While c-MYC was not detectable in freshly isolated articular chondrocytes, as expected, MSC from adipose tissue and bone marrow showed accumulation of c-MYC protein already at passage 0 (P0), which was further upregulated upon cell passaging (P1, P2) (Fig. 1a–c). The abundancy of c-MYC protein in expanded MSC from bone marrow reached higher levels than in the cervical cancer cell line HeLa that is known to induce tumors in a mouse xenograft model [65]. Moreover, MYC protein accumulation stayed high during expansion at later passages (P5 and P9), with comparable levels to those detected at passage 1 (Fig. 1c). The MYC protein accumulation dynamic was in agreement with corresponding data on mRNA expression (Fig. 1d). Additionally, in the absence of basal fibroblast growth factor (bFGF) in the expansion medium, levels of c-MYC dropped already at passage 5, and even further at passage 9 (Fig. 1c), indicating that, indeed, the presence of growth factors in a culture medium supports c-MYC protein accumulation. Since c-MYC is known to play an important role in tumorigenesis as a proto-oncogene, it was important to address next whether high *c-MYC* expression could affect proliferation capacity of the MSC, change their differentiation characteristics, and induce neoplastic transformation.

### *C-MYC* overexpression in MCS correlated with its functional activity

**Fig. 2:**
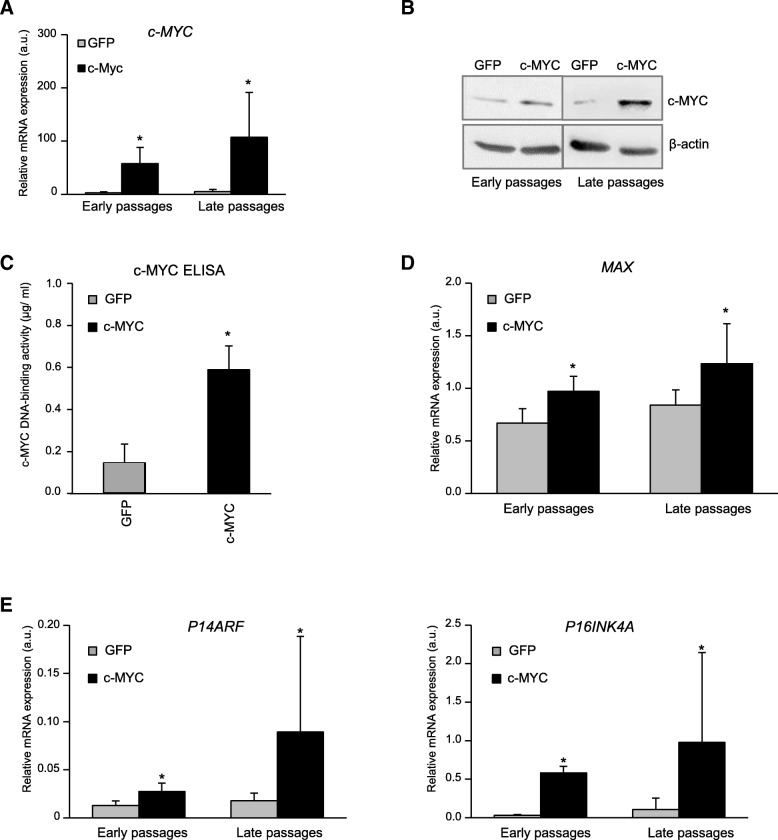
*C-MYC* overexpression in MSC correlated with its functional activity. Bone marrow-derived MSC transduced with either *c-MYC* or *GFP* (control) were expanded and collected at passages 2–4 (early passages) or 9–10 (late passages). **a**, **d**, **e** mRNA expression of indicated genes was measured by qRT-PCR analysis in indicated MSC at early or late passages, as indicated, *n* = 4; *P < 0.05, versus GFP-MSC at similar passages. **b** Western blot analysis of c-MYC protein abundancies in indicated MSC at early or late passages. β-Actin served as a loading control. **c** c-MYC DNA-binding activity was measured by ELISA assay in indicated MSC at passage 10 (*n* = 3) and quantified as the amount of c-MYC (μg/ml) bound to an oligonucleotide containing a c-MYC consensus sequence; *P < 0.05 versus GFP control

To address how *c-MYC* expression affects MSC, we generated MSC with constitutively high *c-MYC* expression. For this, bone marrow MSC were transduced with retrovirus containing the expression cassette for human *c-MYC* (c-MYC-MSC), and a *GFP*-expressing retrovirus was used for obtaining control GFP-MSC. First, we confirmed that after transduction, c-MYC-MSC expressed significantly higher levels of *c-MYC* mRNA in comparison to the control GFP-MSC. Additionally, it remained higher in later passages in comparison to early passages (Fig. 2a), and this effect on mRNA expression correlated with c-MYC protein accumulation (Fig. 2b).

Next, we questioned whether c-MYC protein accumulation due to overexpression also correlated with its function as a transcription factor and whether c-MYC is actively recruited to its consensus sequences at genomic DNA. For this, DNA-binding activity of c-MYC was assessed by TransAM™ ELISA (enzyme-linked immunosorbent assay) applying an antibody specific for the active form of c-MYC when it is bound to its target DNA. We found that, indeed, elevated c-MYC accumulation in c-MYC-MSC led to a significant increase in c-MYC occupancy at its DNA recognition sites in comparison to the control GFP-MSC (Fig. 2c). Additionally, overexpression of *c-MYC* in MSC resulted in elevated expression of its heterodimer binding partner *MAX* (Fig. 2d).

Then, we tested how *c-MYC* overexpression would affect known MYC antagonists, tumor suppressors *P19ARF* and *P16INK4A*, which can block MYC function. We found that expression of both *P14ARF* and *P16INK4A* was significantly elevated in c-MYC-MSC, and it was further increased in later passages by trend (Fig. 2e). We conclude that *c-MYC* overexpression in MSC resulted in subsequent enhancement of biologic functions caused by c-MYC, and these effects became even more pronounced upon ex vivo cell expansion in later passages. However, the observed upregulation of tumor suppressor genes in response to c-MYC activation also suggests that cell safety mechanisms were induced to counteract these effects, as *P16INK4A/P19ARF* expression might prevent a possible neoplastic transformation of MSC by switching on senescence and apoptosis programs.

### *C-MYC* overexpression promoted proliferation of MSC

**Fig. 3:**
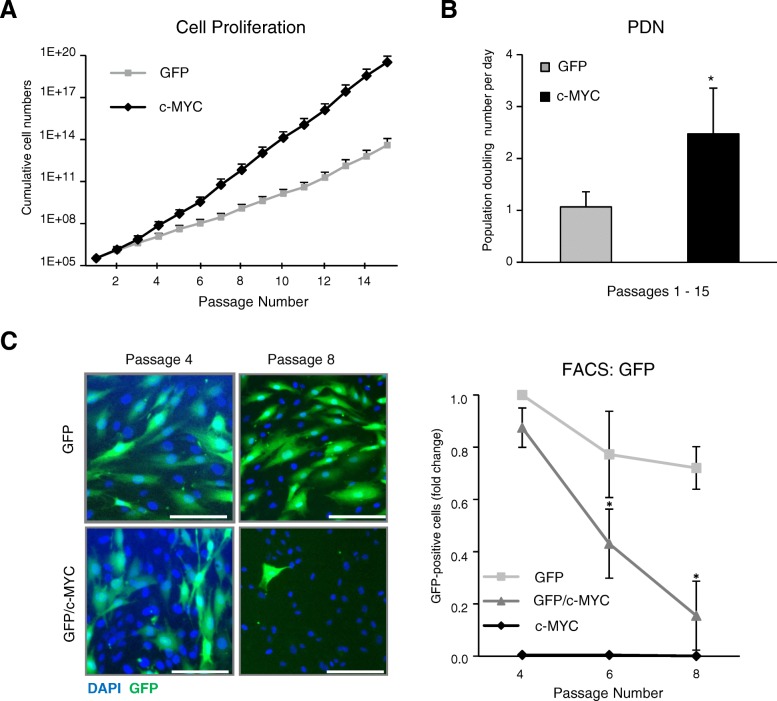
*C-MYC* overexpression promoted proliferation of MSC. Cell proliferation kinetics was assessed by recording cumulative cell numbers (**a**) and the population doubling number per day (PDN) (**b**) in GFP- or c-MYC-MSC at every passage during expansion (passages 1 to 15); *n* = 4; *P < 0.05 (ANOVA test). **c** GFP-MSC and c-MYC-MSC were mixed (4:1, respectively) after passage 4 and co-cultured for four passages. Left: representative images of mixed GFP/c-MYC- or control GFP-MSC at the start of the co-culture (passage 4) and after passage 8. DAPI was used for nuclei staining; Scale bar, 100 μm; right: quantitative analysis of GFP-positive cells in GFP/c-MYC co-cultures, and pure GFP- or c-MYC-MSC cultures, done by flow cytometry. Proportion of GFP-positive cells in corresponding cell populations was quantified in relation to the pure GFP population at passage 4 (set as 1). Asterisks indicate significant differences in comparison to GFP-MSC at passage 4; *n* = 4; *P < 0.05

For many clinical applications, it is important to achieve maximal MSC proliferation and provide sufficient amounts of the cells with multipotent properties. It has been shown before that the total amount of *MYC* transcripts in hematopoietic stem cells (HSC) correlates with the multipotency and self-renewal of these cells [66], so it might be a plausible idea to use *c-MYC* overexpression as an approach to boost MSC proliferation and stemness properties. The effect of c-MYC on the growth and/or differentiation of human MSC has not been clarified yet. Since we found here that the expanded MSC exhibited higher c-MYC levels upon expansion, we hypothesized that c-MYC affects proliferation properties of MSC. To address this question directly, we compared proliferation rates of c-MYC-MSC in comparison to control GFP-expressing ones. For this, we recorded growth curves by counting cell numbers during passages 1 to 15 of MSC derived from four donors. As expected, c-MYC-MSC were proliferating significantly faster than control MSC (GFP-transduced (Fig. 3a) or non-transduced MSC (Additional file 1: Figure S1)), and their population doubling number per day was significantly increased (2.5-fold difference) (Fig. 3b). Of note, there were no significant differences between cell proliferation rates between the two control groups of MSC: GFP and non-transduced (*p* = 0.412; data not shown).

To determine whether higher *c-MYC* expression levels of some MSC may also confer a selection advantage over others during expansion, we examined whether the c-MYC-MSC would overgrow cells with unaltered *c-MYC* expression. For this, after passage 4, these two MSC cell populations were mixed at a following ratio: 80% of GFP-MSC were combined with 20% of c-MYC-MSC, and the cells were co-cultured together for another four passages. In control samples, pure GFP- or c-MYC-MSC cultures were passaged in parallel. It was found that, although the GFP-MSC population comprised a majority in GFP/c-MYC (4:1)-MSC mixed culture at the start of the experiment, four passages later, GFP-positive cells were almost lost from co-culture, and this was not due to loss of the fluorescence signal from GFP, because it was present in control GFP-only MSC at the same passage (Fig. 3c, left). This was supported by quantitative FACS analysis that exhibited a significant decline (12-fold difference) of the GFP-positive MSC population in the co-culture samples within four passages (Fig. 3c, right). Collectively, these data suggest that elevated *c-MYC* expression positively correlated with proliferation capacity of the MSC, and those with higher levels for *c-MYC* were able to overgrow the MSC that had this gene expression unaffected.

### *C-MYC* overexpression reduced osteogenic and adipogenic differentiation of MSC

To find out whether high levels of *c-MYC* would promote or, in contrary, prevent differentiation, we subjected MSC, either overexpressing *c-MYC* or *GFP*, to osteogenic, adipogenic, or chondrogenic differentiation conditions and followed the process by monitoring specific differentiation markers’ dynamics.

**Fig. 4:**
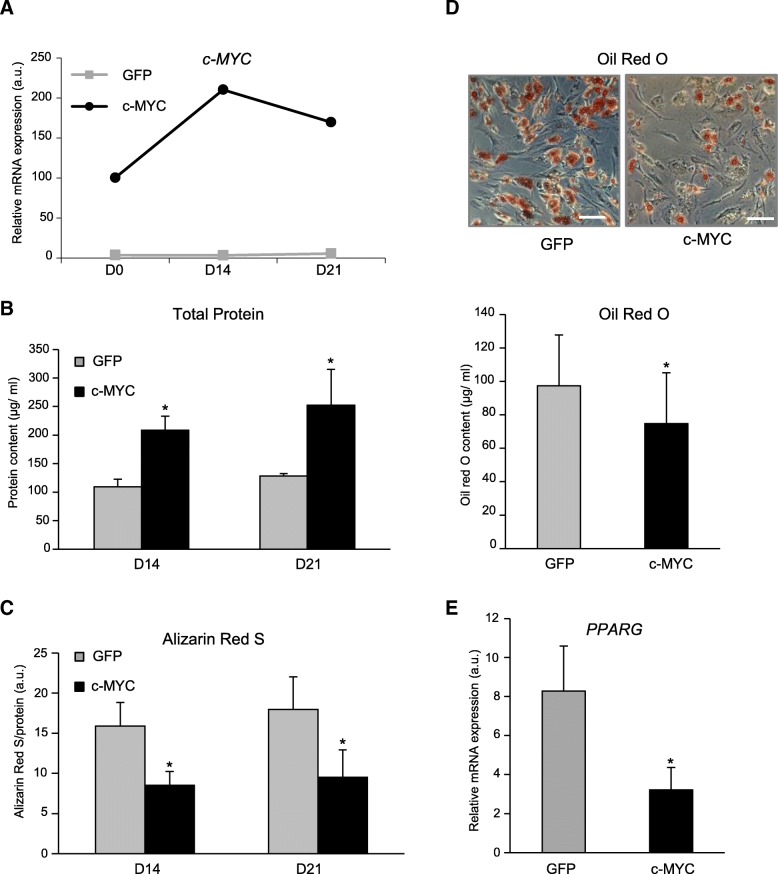
*C-MYC* overexpression reduced osteogenic and adipogenic markers upon MSC differentiation. **a**–**c** c-MYC-MSC or GFP-MSC were expanded to passage 8, and osteogenic differentiation was induced for 3 weeks (D0–D21). **a** *c-MYC* mRNA expression measured by qRT-PCR; *n* = 2. **b** Total protein content monitored with Bradford reagent at day 14. **c** Calcium deposition measured by Alizarin Red S staining and normalized to total protein content; **d**, **e** C-MYC-MSC or GFP-MSC were expanded to passage 8, and adipogenic differentiation was induced for 3 weeks. **d** Top: representative images of MSC stained with Oil Red O; scale bar, 100 μm; bottom: quantitative analysis of Oil Red O content; *n* = 4; *P < 0.05. **b**–**d** MSC were from *n* = 4 donors with combined cells at passages 4, 6, 8, and 10; *P < 0.05, versus GFP control at similar time point. **e** *PPARG* mRNA expression measured by qRT-PCR; *n* = 3, passage 8; *P < 0.05

During a 21-day time-course of osteogenic differentiation, *c-MYC* expression was initially increasing even higher, and only after day 14 showed a tendency to decline. In control GFP-MSC, levels of *c-MYC* stayed low throughout duration of the experiment (with 39-fold difference to the c-MYC-MSC) (Fig. 4a). Higher levels of *c-MYC* during osteogenic differentiation also resulted in significantly higher levels of total protein content during osteogenesis, as a reflection of increased proliferation rates and therefore cell numbers in c-MYC-MSC in comparison to control GFP-MSC (Fig. 4b). To follow progression of the osteogenic differentiation and monitor calcium deposition, we used Alizarin Red S staining. We found that, although both groups displayed successful mineralization, c-MYC-MSC had significantly reduced Alizarin Red S staining per cell at days 14 and 21 (Fig. 4c). Collectively, these results demonstrate that osteogenic differentiation in c-MYC-MSC was reduced.

Next, we followed how *c-MYC* overexpression impacts adipogenesis, and c-MYC- or GFP-MSC were subjected to adipogenic culture conditions after passage 8 for 3 weeks. Although both groups successfully accumulated lipid-containing droplets, c-MYC-MSC had significantly reduced Oil Red O staining comparing to the control GFP-MSC (Fig. 4d). Additionally, the expression of *PPARG* (peroxisome proliferator-activated receptor gamma), an important marker for successful adipogenesis, was significantly diminished in c-MYC-MSC in relation to GFP-labeled cells (Fig. 4e). Therefore, these data suggest that *c-MYC* decreased not only the osteogenic but also the adipogenic potential of MSC.

### *C-MYC* overexpression inhibited chondrogenesis and correlated with increase of hypertrophy

Since we established that *c-MYC* overexpression influenced both osteogenic and adipogenic lineages of human MSC, next, it was important to find out how the chondrogenic differentiation was affected in our model. The main problem for the cartilage regeneration therapy with application of the MSC for joint damage is that the MSC subjected to chondrogenic differentiation undergo highly undesirable hypertrophy resulting in induction of type X collagen, one of the typical markers of endochondral bone formation. In contrary to MSC, articular chondrocytes do not become hypertrophic under the same conditions. They remain collagen type X-negative and can form stable cartilage tissue in immunodeficient mice, while samples from chondrogenically differentiated MSC have been shown to form calcifying grafts [67].

**Fig. 5:**
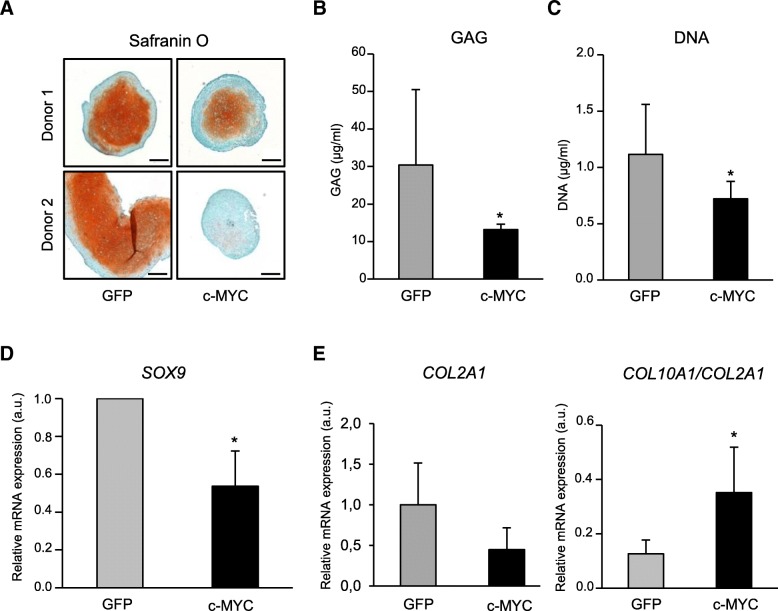
*C-MYC* overexpression inhibited chondrogenesis and correlated with increase of hypertrophy. C-MYC-MSC or GFP-MSC were expanded to passage 4, subjected to chondrogenic differentiation for 6 weeks, and induction of chondrogenic markers was evaluated. **a** Representative images for Safranin O staining in MSC from two selected donors (*n* = 4), as indicated; scale bar, 200 μm. **b** Proteoglycan deposition assessed with DMMB assay; *n* = 3. **c** DNA content quantification done with PicoGreen kit; *n* = 3. **d** *SOX9* mRNA expression measured by qRT-PCR, in relation to GFP control; *n* = 4. **e** *COL2A1* mRNA expression and the ratio of *COL10A1* to *COL2A1* mRNAs were analyzed by qRT-PCR; *n* = 4. *P < 0.05

To address how MYC might impact chondrogenesis, c-MYC- or GFP-MSC were subjected to chondrogenic differentiation after passage 4. Safranin O staining revealed that, similarly to other lineage progressions, chondrogenesis was also affected, as less proteoglycans was deposited in case of c-MYC-MSC in comparison to control GFP-MSC, and overall, c-MYC-MSC formed smaller pellets (Fig. 5a). This was supported by the data on glycosaminoglycan (GAG) deposition that was significantly reduced in case of c-MYC-MSC pellets (Fig. 5b). The same effect was found for DNA content that was also significantly decreased in pellets formed by these cells (Fig. 5c). Additionally, it was accompanied with significantly diminished mRNA expression levels for the main chondrogenic transcription factor *SOX9* (SRY (sex determining region Y)-box 9) in c-MYC-MSC in comparison to GFP-MSC (Fig. 5d). These data were supported by the finding that collagen type II expression (*COL2A1*) also had a trend to decline (Fig. 5e). In contrary to this, the mRNA levels of the hypertrophic marker, collagen type X (*COL10A1*), had a tendency to increase (data not shown). Overall, the ratio between the two collagens, *COL10A1/COL2A1*, was significantly higher in *c-MYC*-overexpressing chondrocytes (Fig. 5e). This finding indicates that c-MYC might also contribute to the onset of hypertrophic differentiation in MSC.

Therefore, all the tested chondrogenic markers uniformly indicated that *c-MYC* overexpression reduced chondrogenic differentiation of the MSC, similarly as it was observed for other lineages. Taken together, these data demonstrate that c-MYC attenuated differentiation of MSC into all three lineages. Additionally, in case of chondrogenesis, *c-MYC* overexpression correlated with increase of collagen type X in relation to the chondrogenesis-enhancing collagen type II. This might also imply a new putative role of c-MYC in promoting a hypertrophic phenotype.

### *C-MYC* overexpression did not interfere with ectopic bone formation by human MSC and caused no aberrant effects in vivo

**Fig. 6:**
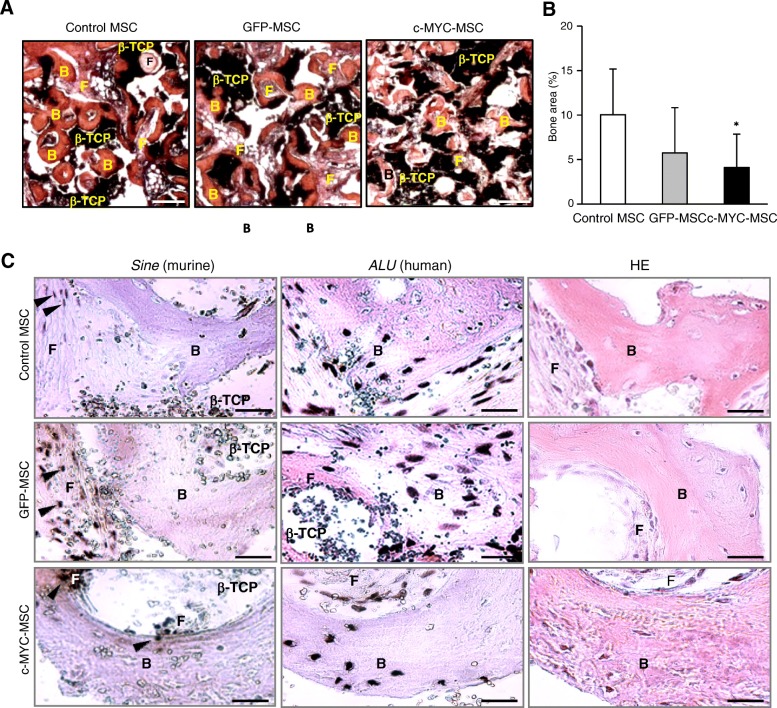
*C-MYC* overexpression did not prevent ectopic bone formation by human MSC in vivo. Bone marrow-derived MSC from five donors were transduced either with c-MYC or GFP, or were left non-transduced (control MSC), then expanded to passage 2 and used for β-TCP/MSC construct preparations which were implanted into SCID mice (*n* = 8) for ectopic bone formation during 6 weeks. **a** Representative images of HE staining of β-TCP explants with corresponding MSC, as indicated; scale bar, 250 μm; **b** histomorphometrical analysis of 48–54 sections for each group; *P < 0.05, versus control non-transduced MSC. **c** In situ hybridization analysis of explanted β-TCP/MSC constructs after ectopic bone formation done in five to six sections for each group, either with a probe specific to human genomic *ALU* sequences or to murine genome repetitive elements *Sine*, as indicated; right: HE staining of corresponding sections indicating ectopic bone positions; black arrows indicate positions of nuclei with positive staining for murine hybridization probe; scale bar, 20 μm. B—ectopic bone tissue; F—fibrous tissue; β-TCP—β-TCP debris

Would *c-MYC* overexpression merely result in decrease of the osteogenic differentiation in vitro or could it abrogate bone formation capacity by MSC implants in vivo? To address this question, as well as follow possible neoplastic transformation induced by the transformed MSC, the regeneration capacity of *c-MYC*-overexpressing MSC was assessed in vivo using an ectopic bone formation mouse model. For this, β-tricalcium phosphate (β-TCP) scaffolds were used as osteoconductive carrier to form implants with MSC which were transplanted ectopically into immunocompromised SCID mice. Most of the tested constructs displayed successful heterotopic bone formation 6 weeks later (with exception of two samples seeded with GFP-MSC from the same donor) (Table 1), and no phenotypic differences for all tested samples were found (Fig. 6a). Histomorphometrical analysis of ectopic bone tissue sections revealed that although the total area of bone tissue derived from c-MYC-MSC constructs in relation to non-transduced control MSC was significantly reduced (2.5-fold), it was comparable to control GFP-MSC (Fig. 6b). Species-specific in situ hybridization confirmed that in every tested MSC group, de novo bone tissue was derived from human MSC and not from mouse cells (Fig. 6c and Additional file 1: Figure S2), thus indicating that *c-MYC* overexpression did not abrogate the bone differentiation program in vivo.

**Table 1:** Ectopic bone formation in β-TCP/MSC constructs (positive samples per total number of explants)

Donor	MSC	GFP-MSC	c-MYC-MSC
A	1/1	1/1	1/1
B	2/2	2/2	2/2
C	2/2	1/1	2/2
D	2/2	2/2	2/2
E	2/2	0/2	2/2
Total	*9/9*	*6/8*	*9/9*

After construct explantation, animals were thoroughly examined for signs of malignant transformation or teratoma growth in their organs and tissues. Macroscopical inspection of liver, lung, spleen, and kidney organs did not indicate a presence of any abnormalities that might suggest tumor formation or malignant transformation processes induced by c-MYC-MSC. Additionally, there was no increase of tissue volume of implants between the tested groups, as well as no evidence for presence of malignant cells in all examined multiple tissue sections. This indicated that higher *c-MYC* expression alone would not immediately lead to tumorigenic transformation of the MSC in vivo.

We conclude that, although *c-MYC* overexpression promoted higher proliferation rates and altered differentiation properties of MSC, no detrimental effects concerning safety of clinical application of these cells in terms of tumorigenesis were detected in the experimental set-up used by this study.

## Discussion

Although MSC represent an attractive cell source for varieties of clinical protocols, their application is restricted due to limited cell numbers available from a bone marrow source. Therefore, there is a requirement for additional expansion of MSC ex vivo before their implantation into a patient. These protocols use culture media containing a mixture of various growth factors necessary for maintaining MSC viability, proliferation, and differentiation potential that also might induce corresponding responsive genes, such as *MYC*. It is known that *MYC* contributes significantly to a range of cellular processes, including proliferation, cell cycle progression, and pluripotency maintenance in both ES and adults stem cells. At the same time, *MYC* is a proto-oncogene, and its link to the tumorigenic potential of cells has been established. Thus, *MYC* plays an ambivalent role as a proliferation and differentiation factor in stem cells, as well as an oncogene in cancer cells. Since no data on the influence of *MYC* on bone marrow-derived MSC were available, it was important to elucidate the effect of aberrant expression of *MYC* on MSC in term of their regenerative properties and safety risks for clinical applications.

Our initial experiments established that, indeed, in expanded MSC, there were high levels of c-MYC protein accumulation, and they further increased with the subsequent passaging of these cells. Additionally, we found that high c-MYC protein levels were sufficiently maintained only in presence of bFGF in the expansion medium. This is in agreement with earlier observations that MYC expression is regulated by FGF signaling [68, 69], as well as with the findings that FGFR1/3 signaling is essential for the maintenance of proliferation and successful chondrogenesis [70]. We hypothesized that *c-MYC* expression is important for the establishment and maintenance of the multipotent stem cell properties of human MSC. Therefore, lack of MYC or the elimination of its function would severely impair the potential of these cells and limit their therapeutic application. On the other side, excessive MYC levels might block differentiation or lead to senescence. We found that, indeed, constitutively elevated expression of *c-MYC* in transduced MSC resulted in a significant increase of their proliferation rates, and *c-MYC* overexpression served as a selection marker enabling the transformed MSC to significantly overgrow the competing control cells in culture. These results were in agreement with previously shown data on ASC demonstrating that *c-MYC* expression correlated with proliferation capacities of these cells, and a decline in *c-MYC* expression, starting at passage 4, reflected in decrease of cell propagation [32]. However, our study is the first one to demonstrate that *c-MYC* expression and proliferative capacity are directly linked in MSC, and high c-MYC levels enable to compete and overgrow the cells with low *c-MYC*.

Remarkably, upon passaging of MSC transduced with *c-MYC*, there was a significant upregulation of MYC antagonist genes, *P16INK4A* and *P14ARF*, suggesting that a safety mechanism switch inducing cellular senescence and preventing neoplastic transformation of MSC was activated. Similar effects have been demonstrated by another study, where initially high expression of *MYC* in ASC, which then attenuated in the later passages, coincided with identical dynamics in the expression pattern of the *CDKN2A* gene [32]. This also might suggest that the ratios between *MYC* and its antagonists, *P14ARF* and *P16INK4A*, together with an evaluation of the duration of their upregulation, might reflect the variability in proliferation capacities between human MSC donors. If so, these parameters could serve as a functional test to assess the quality of donor-derived MSC for therapeutic applications, as those with higher c-MYC levels would grow better.

Although there have been a few studies describing that *MYC* is expressed in MSC during ex vivo expansion, however, there were no evaluation data how this impacts the differentiation potential of human MSC. Therefore, we followed three lineage differentiation processes in MSC transduced with *c-MYC*. It was found that the elevated *c-MYC* expression did not abrogate the differentiation capacity of the MSC, neither in vitro nor in vivo; however, for all three lineages—osteogenic, adipogenic, and chondrogenic—the expression of the corresponding differentiation markers was significantly decreased.

Consequently, a sufficient *c-MYC* expression seems to be essential for high proliferation rates and maintenance of an undifferentiated state of MSC during ex vivo cultivation. It implies that in case of low *c-MYC* expression or loss of its function, MSC might stop proliferating and undergoing differentiation. Indeed, it has been demonstrated earlier in a murine model that elimination of the functional MYC by expression of a dominant negative form induced the differentiation of ES cells [71].

Surprisingly, we found that *c-MYC* overexpression led to an increased *COL10A1/COL2A1* expression ratio, indicating that a balance between these two collagens was shifted towards hypertrophic collagen type X. This leads to a conclusion that c-MYC might be involved in the hypertrophic degeneration process in differentiating MSC. Our previous epigenetic studies in regulatory gene regions of type X collagen revealed characteristic DNA methylation differences between articular chondrocytes and MSC, which increased in the course of MSC differentiation [72]. We have demonstrated there that all possible DNA methylation sites in the *COL10A1* promoter were completely methylated in articular chondrocytes leading to gene silencing, whereas in MSC they remained demethylated at two E-box putative binding sites for the transcription factor MYC. It could be that the increased *COL10A1*/*COL2A1* ratio in the MSC with high *c-MYC* expression is directly linked to increased recruitment of c-MYC to the *COL10A1* promoter that drives a formation of the transcriptional activation complex. This might explain mechanistically how accumulation of c-MYC could lead to a shift towards *COL10A1* expression in our experiments.

Nevertheless, *c-MYC*-overexpressing MSC still could undergo and complete a full osteogenic differentiation program during in vivo ectopic bone formation experiments and successfully form heterotopic bone in recipient animals, although the total volume of de novo bone was reduced. This implies that overexpression of *c*-*MYC* does not abrogate the differentiation program but might affect its correct time-course onset. Indeed, a recent study has demonstrated that *MYC* expression displays an oscillatory pattern which is important for a correct somite segmentation and elongation during embryonic development in mouse [68]. There, it has been hypothesized that *Myc* expression levels regulate and establish a correct timing of WNT and FGF signaling-regulated gene oscillations involved in the segmentation clock of a developing mouse embryo. Our data suggest that a possible attenuation of differentiation observed in vitro may also be found in vivo. However, there was an indication that in case of chondrogenesis, a constitutively high *c-MYC* expression resulted in increase of the hypertrophic marker *COL10A1*. This might suggest that during chondrogenesis there is a critical checkpoint regulated by a correct *c-MYC* expression that defines a switch between proliferation and differentiation programs. It would be interesting to investigate further whether proper timing of different signaling pathways regulating chondrogenesis is linked to MYC activity.

High proliferation rates of MSC with *c-MYC* overexpression observed in this study, together with a reduced differentiation onset, manifest an increase of stem cell-like properties resembled by these cells that may potentiate a risk of tumorigenicity. It is still a matter of on-going debate between researches whether concerns about neoplastic transformation induced by MSC, directly or via their endocrine activity, have solid evidence [31]. By experts in the MSC field, it has been recommended to keep PDN (population doubling number) low to reduce a risk of karyotype changes, although no direct evidence has been provided yet whether ex vivo passaging causes chromosomal aberrations [3, 18].

In this context, we interrogated this question directly and tested the *c-MYC*-overexpressing MSC in vivo to assess whether an increase in stemness and high PDN characteristics observed in these cells would result in tumor growth incidence. Despite of high proliferative potential, differentiation capacity and elevated *c-MYC* expression, the MSC used for implantation into immunodeficient mice did not form any tumors in vivo. No other indications for malignant growth or teratomas (non-malignant tumor-like formations) were observed in our tests.

Different studies on a role of *c-MYC* in malignant transformation suggest that high expression of *MYC* alone is not sufficient to induce malignant transformation, and only when *MYC* gene amplification is combined with other oncogenes overexpression, e.g., *Her2* [49], or with a loss of tumor suppressors, either due to deletion, e.g., *CDKN2A* [50], or loss-of-function, e.g., RB protein [51], this leads to dramatic boost in neoplastic cell growth. Our finding that overexpression of *c-MYC* correlated with *P16INK4A* and *P14ARF* expression suggests that, in case of an absence of the concomitant mutations of other oncogenes and/or tumor suppressors, cells are able to counteract activating functions of c-MYC and induce safety mechanisms. These factors also need to be taken into account during MSC assessment in terms of safety and quality controls of donor material before clinical applications. Additionally, longer observation times need to be included.

Our results are in agreement with many previous reports demonstrating that human MSC and ASC do not cause malignant growth [21–25, 32]. Therefore, in contrary to induced pluripotent stem cells (iPS) cells that are known to induce multiple teratoma growth [73, 74], in case of human MSC, so far, there is not enough evidence to suggest that application of extensively expanded MSC populations which might be enriched in *c-MYC*-overexpressing cells should be hindered by increased tumorigenesis risk. Our data are in line with general conclusions made by the Cell Products Working Party and the Committee for Advanced Therapies elucidating the risk of potential tumorigenicity related to MSC-based therapies [31] agreeing on a conclusion that in current animal models, in which human MSC are used, no direct evidence has been observed to date that would suggest induced tumor formation. As a final remark, it is important to note that this study does not imply that forced *c-MYC* overexpression might be assumed as a probable strategy to boost the proliferation characteristics of MCS for clinical applications. Our data demonstrated that the constituently high expression of c-MYC not only resulted in good proliferation rates of the MSC but also caused a detrimental shift towards a hypertrophic collagen phenotype and cartilage degeneration during chondrogenesis. In addition, to prevent uncontrolled cell proliferation and potential malignant transformation, the intact function of tumor suppressor genes *P14ARF/P16IK4A* regulating apoptosis and senescence would be absolutely essential in selected MSC donors. These two issues point to lack of safety in elevating c-MYC protein levels for therapeutic applications.

## Conclusions

This study demonstrates for the first time that the proliferation capacity of human bone marrow MSC is linked to *c-MYC* expression and suggests a novel putative role of c-MYC in promoting a hypertrophic phenotype during chondrogenesis. Although further investigations might be necessary to assess the risk of tumorigenic transformation that could be caused by application of the MSC undergoing long-term ex vivo expansion, our data suggest that elevated expression of *c-MYC* alone did not immediately lead to tumor formation in the tested in vivo mouse model.

## Additional file

Additional file 1 **Figure S1.** *C-MYC* overexpression promoted proliferation of MSC. Cell proliferation kinetics were assessed by recording cumulative cell numbers in the same-donor MSC, either control non-transduced or c-MYC-transduced, at every passage during expansion (passages 1 to 15); *n* = 3; *P < 0.05 (ANOVA test). Figure S2. Human *ALU* and murine *Sine* probes used for in situ hybridization provide species-specific staining in corresponding bone tissues. In situ hybridization analysis using either mouse or human bone tissue as corresponding positive and negative species controls with probes specific to human genomic *ALU* sequences or to murine genome repetitive elements *Sine*, to demonstrate the absence of cross-reactivity; scale bar, 50 μm. Table S1. List of qRT-PCR primers used in this study. (PDF 341 kb)

## Abbreviations

AC: Articular chondrocytes
ASC: Adipose tissue mesenchymal stromal cells
bFGF: Basic fibroblast growth factor
BMSC: Bone marrow mesenchymal stem cells
DMMB: Dimethyl-methylene Blue
ES: Embryonic stem cells
GAG: Glycosaminoglycan
GvHD: Steroid-refractory graft-versus-host disease
HE: Hematoxylin-eosin
iPS: Induced pluripotent stem cells
MSC: Mesenchymal stromal cells
PDGF: Platelet-derived growth factor
PDN: Population doubling number
β-TCP: Beta-tricalcium phosphate hydrate

## References

[1] Friedenstein AJ, Chailakhyan RK, Gerasimov UV (1987). Bone marrow osteogenic stem cells: in vitro cultivation and transplantation in diffusion chambers. Cell Tissue Kinet.

[2] Winter A, Breit S, Parsch D, Benz K, Steck E, Hauner H, Weber RM, Ewerbeck V, Richter W (2003). Cartilage-like gene expression in differentiated human stem cell spheroids: a comparison of bone marrow-derived and adipose tissue-derived stromal cells. Arthritis Rheum.

[3] Kundrotas G, Gasperskaja E, Slapsyte G, Gudleviciene Z, Krasko J, Stumbryte A, Liudkeviciene R (2016). Identity, proliferation capacity, genomic stability and novel senescence markers of mesenchymal stem cells isolated from low volume of human bone marrow. Oncotarget.

[4] Pittenger MF, Mackay AM, Beck SC, Jaiswal RK, Douglas R, Mosca JD, Moorman MA, Simonetti DW, Craig S, Marshak DR (1999). Multilineage potential of adult human mesenchymal stem cells. Science.

[5] Caplan AI, Dennis JE (2006). Mesenchymal stem cells as trophic mediators. J Cell Biochem.

[6] Andia I, Maffulli N. New biotechnologies for musculoskeletal injuries. Surgeon. 2018. 10.1016/j.surge.2018.08.004. 10.1016/j.surge.2018.08.004 30170915

[7] Jo CH, Lee YG, Shin WH, Kim H, Chai JW, Jeong EC, Kim JE, Shim H, Shin JS, Shin IS (2014). Intra-articular injection of mesenchymal stem cells for the treatment of osteoarthritis of the knee: a proof-of-concept clinical trial. Stem Cells.

[8] Wernicke CM, Grunewald TG, Hendrik J, Kuci S, Kuci Z, Koehl U, Mueller I, Doering M, Peters C, Lawitschka A (2011). Mesenchymal stromal cells for treatment of steroid-refractory GvHD: a review of the literature and two pediatric cases. Int Arch Med.

[9] Chen SL, Fang WW, Ye F, Liu YH, Qian J, Shan SJ, Zhang JJ, Chunhua RZ, Liao LM, Lin S (2004). Effect on left ventricular function of intracoronary transplantation of autologous bone marrow mesenchymal stem cell in patients with acute myocardial infarction. Am J Cardiol.

[10] Agadi S, Shetty AK (2015). Concise review: prospects of bone marrow mononuclear cells and mesenchymal stem cells for treating status epilepticus and chronic epilepsy. Stem Cells.

[11] Caplan AI (2009). Why are MSCs therapeutic? New data: new insight. J Pathol.

[12] Dexheimer V, Mueller S, Braatz F, Richter W (2011). Reduced reactivation from dormancy but maintained lineage choice of human mesenchymal stem cells with donor age. PLoS One.

[13] Ryan JM, Barry FP, Murphy JM, Mahon BP (2005). Mesenchymal stem cells avoid allogeneic rejection. J Inflammation (London).

[14] Di Trapani M, Bassi G, Midolo M, Gatti A, Takam Kamga P, Cassaro A, Carusone R, Adamo A, Krampera M (2016). Differential and transferable modulatory effects of mesenchymal stromal cell-derived extracellular vesicles on T, B and NK cell functions. Sci Rep.

[15] De Miguel MP, Fuentes-Julian S, Blazquez-Martinez A, Pascual CY, Aller MA, Arias J, Arnalich-Montiel F (2012). Immunosuppressive properties of mesenchymal stem cells: advances and applications. Curr Mol Med.

[16] Samuelsson H, Ringden O, Lonnies H, Le Blanc K (2009). Optimizing in vitro conditions for immunomodulation and expansion of mesenchymal stromal cells. Cytotherapy.

[17] Turinetto V, Vitale E, Giachino C (2016). Senescence in human mesenchymal stem cells: functional changes and implications in stem cell-based therapy. Int J Mol Sci.

[18] Stultz BG, McGinnis K, Thompson EE, Lo Surdo JL, Bauer SR, Hursh DA (2016). Chromosomal stability of mesenchymal stromal cells during in vitro culture. Cytotherapy.

[19] De Witte SFH, Peters FS, Merino A, Korevaar SS, Van Meurs JBJ, O'Flynn L, Elliman SJ, Newsome PN, Boer K, Baan CC (2018). Epigenetic changes in umbilical cord mesenchymal stromal cells upon stimulation and culture expansion. Cytotherapy.

[20] Wang Y, Han ZB, Song YP, Han ZC (2012). Safety of mesenchymal stem cells for clinical application. Stem Cells Int.

[21] Avanzini MA, Bernardo ME, Cometa AM, Perotti C, Zaffaroni N, Novara F, Visai L, Moretta A, Del Fante C, Villa R (2009). Generation of mesenchymal stromal cells in the presence of platelet lysate: a phenotypic and functional comparison of umbilical cord blood- and bone marrow-derived progenitors. Haematologica.

[22] Conforti A, Starc N, Biagini S, Tomao L, Pitisci A, Algeri M, Sirleto P, Novelli A, Grisendi G, Candini O (2016). Resistance to neoplastic transformation of ex-vivo expanded human mesenchymal stromal cells after exposure to supramaximal physical and chemical stress. Oncotarget.

[23] Centeno C, Markle J, Dodson E, Stemper I, Williams CJ, Hyzy M, Ichim T, Freeman M (2017). Treatment of lumbar degenerative disc disease-associated radicular pain with culture-expanded autologous mesenchymal stem cells: a pilot study on safety and efficacy. J Transl Med.

[24] Usha L, Rao G, Christopherson Ii K, Xu X (2013). Mesenchymal stem cells develop tumor tropism but do not accelerate breast cancer tumorigenesis in a somatic mouse breast cancer model. PLoS One.

[25] Bernardo ME, Zaffaroni N, Novara F, Cometa AM, Avanzini MA, Moretta A, Montagna D, Maccario R, Villa R, Daidone MG (2007). Human bone marrow derived mesenchymal stem cells do not undergo transformation after long-term in vitro culture and do not exhibit telomere maintenance mechanisms. Cancer Res.

[26] Miura M, Miura Y, Padilla-Nash HM, Molinolo AA, Fu B, Patel V, Seo BM, Sonoyama W, Zheng JJ, Baker CC (2006). Accumulated chromosomal instability in murine bone marrow mesenchymal stem cells leads to malignant transformation. Stem Cells.

[27] Tsukamoto S, Honoki K, Fujii H, Tohma Y, Kido A, Mori T, Tsujiuchi T, Tanaka Y (2012). Mesenchymal stem cells promote tumor engraftment and metastatic colonization in rat osteosarcoma model. Int J Oncol.

[28] Houghton J, Stoicov C, Nomura S, Rogers AB, Carlson J, Li H, Cai X, Fox JG, Goldenring JR, Wang TC (2004). Gastric cancer originating from bone marrow-derived cells. Science.

[29] Suva ML, Riggi N, Stehle JC, Baumer K, Tercier S, Joseph JM, Suva D, Clement V, Provero P, Cironi L (2009). Identification of cancer stem cells in Ewing’s sarcoma. Cancer Res.

[30] Cironi L, Provero P, Riggi N, Janiszewska M, Suva D, Suva ML, Kindler V, Stamenkovic I (2009). Epigenetic features of human mesenchymal stem cells determine their permissiveness for induction of relevant transcriptional changes by SYT-SSX1. PLoS One.

[31] Barkholt L, Flory E, Jekerle V, Lucas-Samuel S, Ahnert P, Bisset L, Buscher D, Fibbe W, Foussat A, Kwa M (2013). Risk of tumorigenicity in mesenchymal stromal cell-based therapies--bridging scientific observations and regulatory viewpoints. Cytotherapy.

[32] Paula AC, Martins TM, Zonari A, Frade SP, Angelo PC, Gomes DA, Goes AM (2015). Human adipose tissue-derived stem cells cultured in xeno-free culture condition enhance c-MYC expression increasing proliferation but bypassing spontaneous cell transformation. Stem Cell Res Ther.

[33] Cosgrave N, Hill AD, Young LS (2006). Growth factor-dependent regulation of survivin by c-myc in human breast cancer. J Mol Endocrinol.

[34] Chiariello M, Marinissen MJ, Gutkind JS (2001). Regulation of c-myc expression by PDGF through Rho GTPases. Nat Cell Biol.

[35] Hu MC, Rosenblum ND (2005). Smad1, beta-catenin and Tcf4 associate in a molecular complex with the Myc promoter in dysplastic renal tissue and cooperate to control Myc transcription. Development.

[36] Kumamoto M, Nishiwaki T, Matsuo N, Kimura H, Matsushima K (2009). Minimally cultured bone marrow mesenchymal stem cells ameliorate fibrotic lung injury. Eur Respir J.

[37] Chen B, Yu J, Wang Q, Zhao Y, Sun L, Xu C, Zhao X, Shen B, Wang M, Xu W (2018). Human bone marrow mesenchymal stem cells promote gastric cancer growth via regulating c-Myc. Stem Cells Int.

[38] Beltran H (2014). The N-myc oncogene: maximizing its targets, regulation, and therapeutic potential. Mol Cancer Res.

[39] Ikegaki N, Minna J, Kennett RH (1989). The human L-myc gene is expressed as two forms of protein in small cell lung carcinoma cell lines: detection by monoclonal antibodies specific to two myc homology box sequences. EMBO J.

[40] Luscher B (2001). Function and regulation of the transcription factors of the Myc/Max/Mad network. Gene.

[41] Qi Y, Gregory MA, Li Z, Brousal JP, West K, Hann SR (2004). p19ARF directly and differentially controls the functions of c-Myc independently of p53. Nature.

[42] Abida WM, Gu W (2008). p53-Dependent and p53-independent activation of autophagy by ARF. Cancer Res.

[43] Shibata KR, Aoyama T, Shima Y, Fukiage K, Otsuka S, Furu M, Kohno Y, Ito K, Fujibayashi S, Neo M (2007). Expression of the p16INK4A gene is associated closely with senescence of human mesenchymal stem cells and is potentially silenced by DNA methylation during in vitro expansion. Stem Cells.

[44] Nesbit CE, Tersak JM, Prochownik EV (1999). MYC oncogenes and human neoplastic disease. Oncogene.

[45] Gabay M, Li Y, Felsher DW. MYC activation is a hallmark of cancer initiation and maintenance. Cold Spring Harb Perspect Med. 2014;4(6):a014241. 10.1101/cshperspect.a014241. 10.1101/cshperspect.a014241 PMC4031954 24890832

[46] Cascon A, Robledo M (2012). MAX and MYC: a heritable breakup. Cancer Res.

[47] Wolfer A, Ramaswamy S (2011). MYC and metastasis. Cancer Res.

[48] Knoepfler PS (2008). Why myc? An unexpected ingredient in the stem cell cocktail. Cell Stem Cell.

[49] Nair R, Roden DL, Teo WS, McFarland A, Junankar S, Ye S, Nguyen A, Yang J, Nikolic I, Hui M (2014). c-Myc and Her2 cooperate to drive a stem-like phenotype with poor prognosis in breast cancer. Oncogene.

[50] Shimizu T, Ishikawa T, Sugihara E, Kuninaka S, Miyamoto T, Mabuchi Y, Matsuzaki Y, Tsunoda T, Miya F, Morioka H (2010). c-MYC overexpression with loss of Ink4a/Arf transforms bone marrow stromal cells into osteosarcoma accompanied by loss of adipogenesis. Oncogene.

[51] Wang JY, Wu PK, Chen PC, Lee CW, Chen WM, Hung SC (2017). Generation of osteosarcomas from a combination of Rb silencing and c-Myc overexpression in human mesenchymal stem cells. Stem Cells Transl Med.

[52] Gandarillas A, Watt FM (1997). c-Myc promotes differentiation of human epidermal stem cells. Genes Dev.

[53] Watt FM, Frye M, Benitah SA (2008). MYC in mammalian epidermis: how can an oncogene stimulate differentiation?. Nat Rev Cancer.

[54] Kim DS, Ko YJ, Lee MW, Park HJ, Park YJ, Kim DI, Sung KW, Koo HH, Yoo KH (2016). Effect of low oxygen tension on the biological characteristics of human bone marrow mesenchymal stem cells. Cell Stress Chaperones.

[55] Heo JS, Choi Y, Kim HS, Kim HO (2016). Comparison of molecular profiles of human mesenchymal stem cells derived from bone marrow, umbilical cord blood, placenta and adipose tissue. Int J Mol Med.

[56] Bothe F, Lotz B, Seebach E, Fischer J, Hesse E, Diederichs S, Richter W (2018). Stimulation of calvarial bone healing with human bone marrow stromal cells versus inhibition with adipose-tissue stromal cells on nanostructured beta-TCP-collagen. Acta Biomater.

[57] Praxenthaler H, Krämer E, Weisser M, Hecht N, Fischer J, Grossner T, Richter W (2018). Extracellular matrix content and WNT/β-catenin levels of cartilage determine the chondrocyte response to compressive load. Biochim Biophys Acta (BBA) - Mol Basis Dis.

[58] Dexheimer V, Gabler J, Bomans K, Sims T, Omlor G, Richter W (2016). Differential expression of TGF-beta superfamily members and role of Smad1/5/9-signalling in chondral versus endochondral chondrocyte differentiation. Sci Rep.

[59] Takahashi K, Tanabe K, Ohnuki M, Narita M, Ichisaka T, Tomoda K, Yamanaka S (2007). Induction of pluripotent stem cells from adult human fibroblasts by defined factors. Cell.

[60] Willemsen RA, Weijtens ME, Ronteltap C, Eshhar Z, Gratama JW, Chames P, Bolhuis RL (2000). Grafting primary human T lymphocytes with cancer-specific chimeric single chain and two chain TCR. Gene Ther.

[61] Anton M, Wagner B, Haubner R, Bodenstein C, Essien BE, Bonisch H, Schwaiger M, Gansbacher B, Weber WA (2004). Use of the norepinephrine transporter as a reporter gene for non-invasive imaging of genetically modified cells. J Gene Med.

[62] Rao X, Huang X, Zhou Z, Lin X (2013). An improvement of the 2^(-delta delta CT) method for quantitative real-time polymerase chain reaction data analysis. Biostat Bioinforma Biomath.

[63] Bohner M, van Lenthe GH, Grunenfelder S, Hirsiger W, Evison R, Muller R (2005). Synthesis and characterization of porous beta-tricalcium phosphate blocks. Biomaterials.

[64] Steck E, Burkhardt M, Ehrlich H, Richter W (2010). Discrimination between cells of murine and human origin in xenotransplants by species specific genomic in situ hybridization. Xenotransplantation.

[65] Li HN, Nie FF, Liu W, Dai QS, Lu N, Qi Q, Li ZY, You QD, Guo QL (2009). Apoptosis induction of oroxylin A in human cervical cancer HeLa cell line in vitro and in vivo. Toxicology.

[66] Laurenti E, Varnum-Finney B, Wilson A, Ferrero I, Blanco-Bose WE, Ehninger A, Knoepfler PS, Cheng PF, MacDonald HR, Eisenman RN (2008). Hematopoietic stem cell function and survival depend on c-Myc and N-Myc activity. Cell Stem Cell.

[67] Pelttari K, Winter A, Steck E, Goetzke K, Hennig T, Ochs BG, Aigner T, Richter W (2006). Premature induction of hypertrophy during in vitro chondrogenesis of human mesenchymal stem cells correlates with calcification and vascular invasion after ectopic transplantation in SCID mice. Arthritis Rheum.

[68] Mastromina I, Verrier L, Silva JC, Storey KG, Dale JK. Myc activity is required for maintenance of the neuromesodermal progenitor signalling network and for segmentation clock gene oscillations in mouse. Development. 2018;145(14):dev161091. 10.1242/dev.161091. 10.1242/dev.161091 PMC6078331 30061166

[69] Dombrowski C, Helledie T, Ling L, Grunert M, Canning CA, Jones CM, Hui JH, Nurcombe V, van Wijnen AJ, Cool SM (2013). FGFR1 signaling stimulates proliferation of human mesenchymal stem cells by inhibiting the cyclin-dependent kinase inhibitors p21(Waf1) and p27(Kip1). Stem Cells.

[70] Fischer J, Knoch N, Sims T, Rosshirt N, Richter W (2018). Time-dependent contribution of BMP, FGF, IGF, and HH signaling to the proliferation of mesenchymal stroma cells during chondrogenesis. J Cell Physiol.

[71] Cartwright P, McLean C, Sheppard A, Rivett D, Jones K, Dalton S (2005). LIF/STAT3 controls ES cell self-renewal and pluripotency by a Myc-dependent mechanism. Development.

[72] Zimmermann P, Boeuf S, Dickhut A, Boehmer S, Olek S, Richter W (2008). Correlation of COL10A1 induction during chondrogenesis of mesenchymal stem cells with demethylation of two CpG sites in the COL10A1 promoter. Arthritis Rheum.

[73] Prokhorova TA, Harkness LM, Frandsen U, Ditzel N, Schroder HD, Burns JS, Kassem M (2009). Teratoma formation by human embryonic stem cells is site dependent and enhanced by the presence of Matrigel. Stem Cells Dev.

[74] Yasuda S, Kusakawa S, Kuroda T, Miura T, Tano K, Takada N, Matsuyama S, Matsuyama A, Nasu M, Umezawa A (2018). Tumorigenicity-associated characteristics of human iPS cell lines. PLoS One.

