# Supplementary material for: Impact of c-MYC expression on proliferation, differentiation, and risk of neoplastic transformation of human mesenchymal stromal cells
Source: Stem Cell Res Ther. 2019 Mar 5;10:73. doi: 10.1186/s13287-019-1187-z (PMC6402108; doi:10.1186/s13287-019-1187-z)
Supplement: Supplementary file 1 — Figure S1. C-MYC overexpression promoted proliferation of MSC. Cell proliferation kinetics were assessed by recording cumulative cell numbers in the same-donor MSC, either control non-transduced or c-MYC-transduced, at every passage during expansion (passages 1 to 15); n = 3; *P < 0.05 (ANOVA test). Figure S2. Human ALU and murine Sine probes used for in situ hybridization provide species-specific staining in corresponding bone tissues. In situ hybridization analysis using either mouse or human bone tissue as corresponding positive and negative species controls with probes specific to human genomic ALU sequences or to murine genome repetitive elements Sine, to demonstrate the absence of cross-reactivity; scale bar, 50 μm. Table S1. List of qRT-PCR primers used in this study. (PDF 341 kb) [file 13287_2019_1187_MOESM1_ESM.pdf]

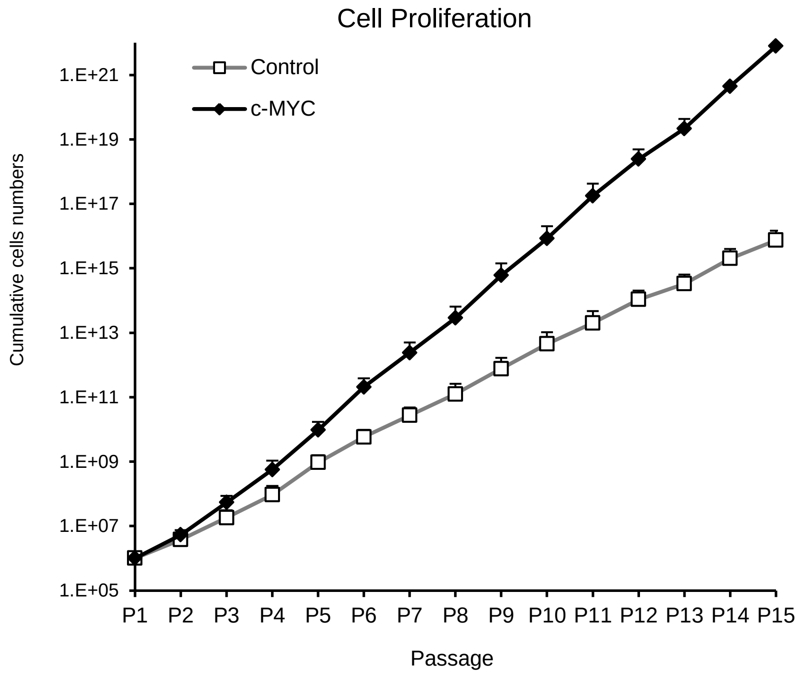

Additional Figure 1. *C-MYC* overexpression promoted proliferation of MSC. Cell proliferation kinetics were assessed by recording cumulative cell numbers in the same-donor MSC, either control non-transduced or c-MYC-transduced, at every passage during expansion (passages 1 to 15); n = 3; *$P < 0.05$ (ANOVA test).
